# Secondary Neuroendocrine Carcinoma Following High-Dose Radiotherapy for Head and Neck Cancer: Report of Two Cases

**DOI:** 10.7759/cureus.847

**Published:** 2016-10-25

**Authors:** Amandeep S Taggar, Roderick Simpson, Desiree Hao, Marc Webster, Moosa Khalil, John Lysack, David Skarsgard

**Affiliations:** 1 Radiation Oncology, Memorial Sloan-Kettering Cancer Center; 2 Radiation Oncology, University of Calgary/Tom Baker Cancer Center; 3 Department of Pathology, University of Calgary; 4 Medical Oncology, University of Calgary; 5 Department of Diagnostic Imaging, University of Calgary

**Keywords:** secondary cancer, radiation, head and neck, neuroendocrine

## Abstract

Patients treated with radiation have an increased risk of developing second cancers, of which carcinomas, sarcomas, and hematological malignancies have most commonly been reported. Neuroendocrine carcinomas (NEC) are rarely reported in patients previously treated with radiation. Two patients, who had successfully undergone chemoradiotherapy for head and neck cancers at our institution, developed secondary NEC within the radiation field more than five years after the treatment. Both patients underwent curative-intent treatment of secondary malignancies, one with chemotherapy, radiation and surgery (Case 1) and the other with chemotherapy and surgery (Case 2). Both had no evidence of disease at a short follow-up of twelve months (Case 1), and three months (Case 2) after treatment. NEC can develop post-radiotherapy; a multidisciplinary approach is necessary to successfully treat these patients.

## Introduction

Radiation has long been recognized to have carcinogenic potential, as shown by longitudinal research on A-bomb survivors of Hiroshima and Nagasaki [1]. Longitudinal studies of cancer survivors, initially treated with radiotherapy, also report an increased risk of secondary malignant neoplasms [2-3]. Most commonly reported second malignancies include carcinomas and hematologic cancers in the low-dose areas, and sarcomas in the high-dose areas [4].

Primary neuroendocrine carcinoma (NEC) most commonly occurs in the gastrointestinal tract or lungs; however, cases of primary head and neck NEC have also been reported [5]. The development of secondary NEC (sNEC) within a previous radiation field has rarely been reported. We found a single report describing one patient in whom sNEC developed ten years after the initial treatment of head and neck squamous cell carcinoma (SCC) [6]. The patient was successfully treated with multimodality treatment. Herein we describe two such cases treated at our institution.

## Case presentation

### Case 1

A 40-year-old male of Asian descent, non-smoker, was diagnosed in 2007 with Stage III (T2bN2M0) (AJCC 6th edition) EBV encoded RNA (EBER) positive nasopharyngeal undifferentiated carcinoma. The patient completed curative-intent concurrent chemo-radiation therapy (70 Gy in 33 fractions to a high-dose volume, including nasopharynx and bilateral-involved neck nodes, and 59.4 Gy in 33 fractions to at-risk areas around the primary site and bilateral neck), three cycles of cisplatin 100mg/m2, given as a bolus q3week), and adjuvant chemotherapy (three cycles of cisplatin and 5-fluorouracil).

**Figure 1:**
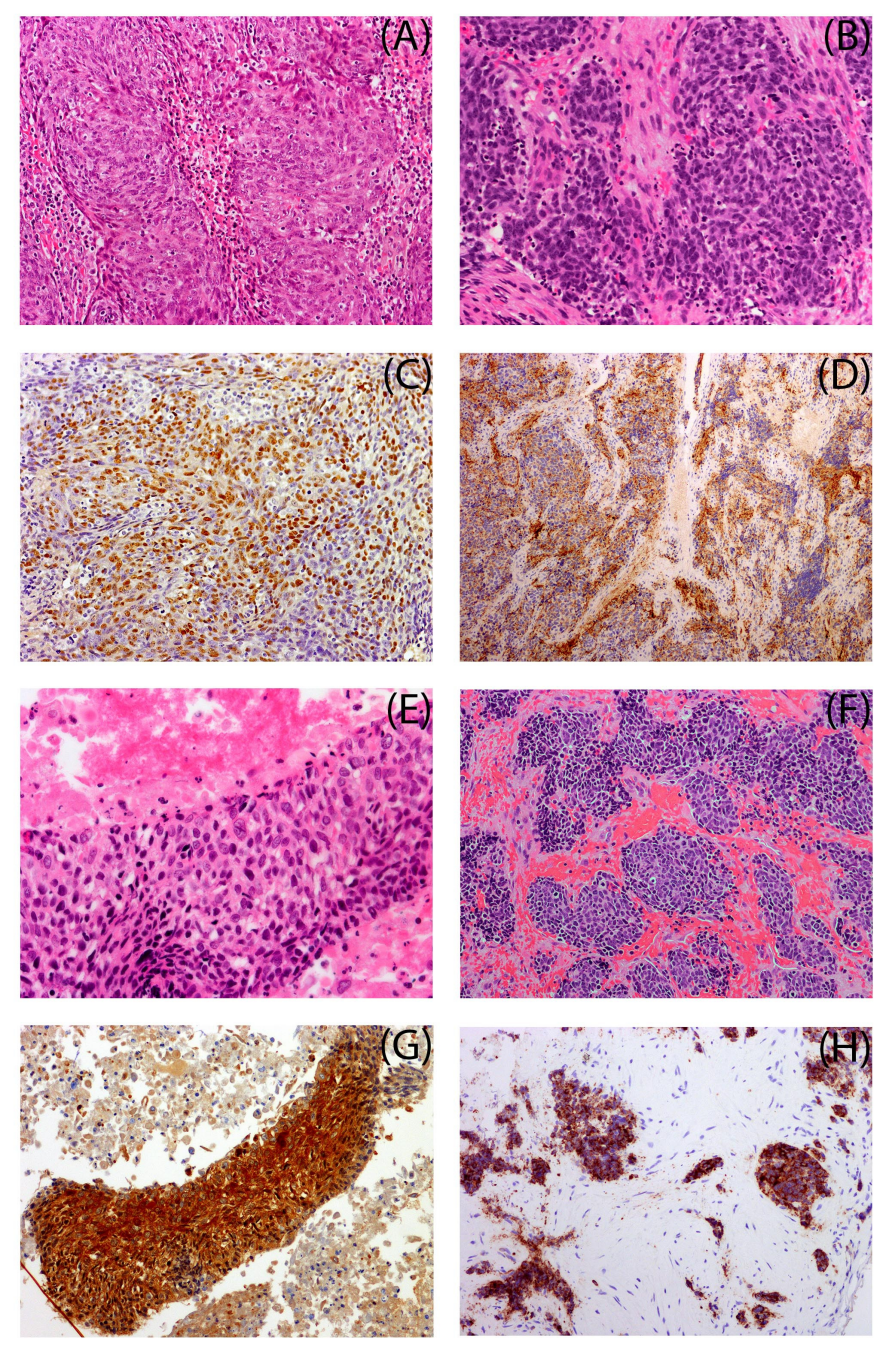
Immunohistochemical Stains of Primary and Secondary Malignancies of Two Cases Case 1 (panels A – D) and Case 2 (panels E – H). Panel A – hematoxylin and eosin (H&E) stained section of nasopharyngeal SCC diagnosed in 2007, and Panel C shows positive EBER in-situ hybridization confirming that this malignancy is related to the Epstein Barr virus (EBV). Panel B – H&E stained section shows poorly differentiated neuroendocrine carcinoma diagnosed in Case 1 in 2013, and panel D shows positive synaptophysin immunohistochemistry confirming the neuroendocrine differentiation of the neoplastic cells. Panel E – H&E stained section of the lymph node core biopsy of Case 2 shows SCC from 2008, and panel G shows positive p16 immunohistochemical staining in SCC indicating HPV etiology. Panel F – H&E stained section shows small cells of neuroendocrine carcinoma, and panel H shows positive synaptophysin immunohistochemistry confirming neuroendocrine differentiation in the second malignancy of 2015.

**Figure 2:**
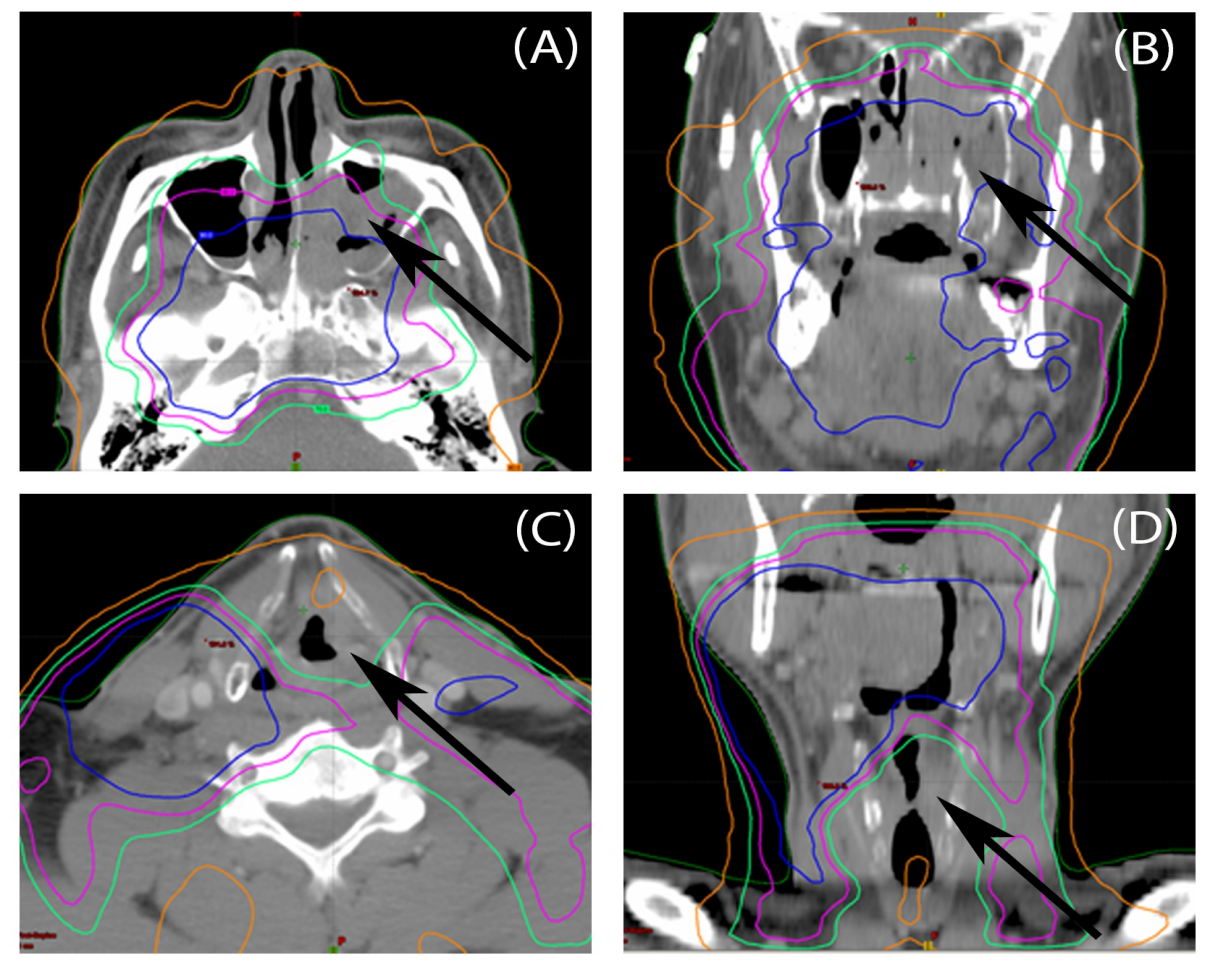
Location of Secondary Malignancy in Relation to Prior Radiation Field for Two Cases CT images of NEC tumors (arrows) in Case 1: Panels A & B, a heterogeneously enhancing polypoid lesion fills the posterior nasal cavity and extends into the maxillary sinus on the left. Case 2: Panels C & D, an enhancing lesion involves the left supraglottic larynx at the level of the false cord, with invasion of the paraglottic space but no destruction of the thyroid cartilage. Isodose lines (blue-90%, purple-80%, green-70% and yellow-40%) from previous treatments are superimposed on diagnostic CT scans, after fusing them in the treatment planning system.

Five years post-treatment, he had no evidence of disease and was placed on annual follow-up. In between follow-up appointments, he presented to a local emergency department with confusion, hypertension, and hyponatremia; and was diagnosed with syndrome of inappropriate ADH (SIADH). A CT scan and MRI of the head at that time showed a polypoid lesion in the left posterior nasal cavity. After being reviewed by an expert head and neck pathologist (MK), this tumor was felt to be distinctly different and demonstrated high-grade NEC that was synaptophysin positive and EBER negative. This tumor was distinctly different from the initial undifferentiated nasopharyngeal carcinoma treated in 2007. Figures 1A-1D show histological differences between the two malignancies. Figures 2A-2B show the location of the secondary tumor in relation to the previous radiation dose distribution.

An MRI of the brain showed no intracranial extension. PET CT scans did not identify the presence of any other primary tumors and confirmed that this was not a metastasis. A sinonasal surgeon was consulted, and his tumor was deemed inoperable.

The patient was treated with platinum/etoposide chemotherapy. An interim PET/CT after three cycles of chemotherapy showed a residual mass, which remained intensely FDG avid even after completing all six cycles. On further discussion with the surgeon, resection was still not recommended.

After full discussion of the risks/benefits of re-irradiation, the patient received consolidative curative-intent radiotherapy to PET-positive areas within the nasal cavity and nasopharynx. A dose of 60 Gy in 30 fractions was delivered to the residual nodule with a 1 cm margin to the cerebral venous sinus thrombosis (CTV), while the prechemotherapy tumor extent and limited bilateral upper cervical and retropharyngeal nodal regions received 50 Gy in 30 fractions. Clinical exam and PET/CT three-months post-completion of radiotherapy treatment showed a small, residual FDG-avid mass in the left nasal antrum which was then surgically resected. Pathology confirmed residual neuroendocrine carcinoma with negative resection margins.

Outcome – Twelve months after completion of the treatment, the patient has no clinical or radiological evidence of disease on repeat PET and MR imaging. He has developed Grade 2 dysarthria (CTCAE v4), but has not developed any other significant late toxicities.  

### Case 2

A 44-year-old Caucasian male, non-smoker, was diagnosed in 2008 with Stage IVA (TxN2bM0) (AJCC 6th edition) p16 positive SCC of the head and neck of unknown primary origin. The patient was treated with radical radiotherapy plus concurrent chemotherapy (2 cycles of 100mg/m2 cisplatin, given as a bolus q3week). An area including grossly involved lymph nodes in the right neck received curative-intent 70 Gy in 33 fractions, while the electively treated bilateral neck and likely primary sites in the oropharynx received 59.4 Gy in 33 fractions. The larynx received between 40% and 50% of the prescribed 70 Gy dose. The patient had no clinical or radiological evidence of recurrent disease five years post-treatment.  

Six and a half years after the initial diagnosis, the patient developed voice loss. A nasopharyngoscopic exam showed a smooth, left supraglottic mass which prevented good glottic closure. Biopsy of this mass showed poorly differentiated NEC. Figures 1E-1H show the histological differences between the two malignancies. Figures 2C-2D show the location of the second primary tumor in relation to the previous radiation dose distribution. CT scans of the head, neck, chest, abdomen, and pelvis, as well as a bone scan, showed no evidence of disease elsewhere. The patient was treated with four cycles of cisplatin and etoposide. A restaging PET/CT showed complete radiographic remission. The patient subsequently underwent hemi-laryngectomy which showed only a microscopic focus (0.2 cm) of residual tumor involving the left true vocal cord with negative resection margins.

Outcome – Three months after completion of the treatment, the patient has no clinical evidence of disease on laryngoscopy. The patient, however, continues to suffer from poor voice and mild difficulty swallowing.

Both patients agreed to participate and were explained the nature and objectives of this study, and informed consents were formally obtained. No references to the patients' identities were made at any stage during data analysis or in the report.

## Discussion

Secondary cancer is a rare, but important sequela of ionizing radiation used to treat cancer. In order to consider a malignancy as having been radiation-induced, a tumor must fulfill the Cahan criteria, i.e., it must i) have temporal relation with radiation, with latency of at least five years, ii) occur within or close to previous radiation fields or beam pathways, and iii) have a different histology [7]. Table 1 shows the histologic differences between primary and secondary tumors in our two cases. The reported incidence of secondary cancers ranges between 0.5% and 15% at 15 years in adult and pediatric populations, respectively [2-3, 8]. The most commonly reported secondary cancers include gastric, colon, breast, and thyroid carcinomas; but secondary cancers in the head and neck have also been reported, for example, by Friedman et al [8]. They stratified radiation-induced cancers based on the anatomic site and found that the rate of secondary head and neck cancer was 0.1% with a median latency period of 7.8 years. In our two patients, the time to onset of their secondary malignancy was 5.7 and 6.4 years, respectively.

Primary NEC of the head and neck is a rare entity and secondary NEC is even more unusual. We report two cases of radiation-induced secondary NEC arising in the head and neck, which are both no evidence of disease (NED) on short-term follow-up after multi-modality treatment of the secondary cancer. Treatment of secondary cancer is particularly challenging, though cure may still be possible. The intense treatments necessary to achieve a cure may lead to increased toxicity and fatal morbidity. A high incidence of Grade 3 or higher morbidity is seen in patients re-irradiated for recurrent or second primary tumors of the head and neck [9]. Therefore, multidisciplinary involvement to tailor treatments to the individual patient is important. Two-year overall and metastasis-free survival rates are reported to be 68% and 40%, respectively, with combined modality treatment for primary head and neck NEC [10]. Data on outcomes of secondary NEC are scarce, and the only case report had a short follow-up (approximately 12 months) [6]. Therefore, our report adds to the limited body of literature for this rare phenomenon.

**Table 1:** Histological and Immunohistochemical Profiles of Initial and Secondary Malignancies in Our Two Cases. SCC – squamous cell carcinoma, NEC – neuroendocrine carcinoma, CK – cytokeratin, EBER – EBV encoded RNA, TTF – thyroid transcription factor ND – not done, +++ strongly positive, ++ positive, - negative, (F+) focally positive, *part of 3ABE12 stain showing strong positivity

	Case 1		Case 2	
Stain	1^st^ malignancy SCC	2^nd^ malignancy NEC	1^st^ malignancy SCC	2^nd^ malignancy NEC
CK7	-	-	-	+++
CK20	ND	ND	-	-
CK5/6	+++	-	+++*	-
Synaptophysin	-	++	-	+++
P63	+++	-	ND	-
EBER	++	-	ND	ND
P16	ND	ND	+++	(F+)
TTF-1	ND	(F+)	-	(F+)

## Conclusions

Development of secondary cancer is an established, long-term risk after initial radiotherapy. Patients should be informed of this risk at the time of initial discussion concerning treatment. Careful follow-up is paramount in patients undergoing radiation to not only assess treatment complications but also to survey for development of secondary cancers. Multi-disciplinary management is essential for patients presenting with secondary cancer within previously irradiated fields.
